# Views on wet nursing and expressing breastmilk for sharing and human milk bank donation among mothers in two parenting social media communities in Vietnam

**DOI:** 10.1111/mcn.13694

**Published:** 2024-08-15

**Authors:** Tuan T. Nguyen, Ngoc L. Huynh, Genevieve Becker, Hoang T. Tran, Jennifer Cashin, Roger Mathisen

**Affiliations:** ^1^ Alive & Thrive, Global Nutrition, FHI 360 Hanoi Vietnam; ^2^ Keck School of Medicine University of Southern California Los Angeles California USA; ^3^ Social Marketing and Communication (SMC), FHI 360 Washington, DC USA; ^4^ BEST Services Galway Ireland; ^5^ Neonatal Unit and Human Milk Bank, Da Nang Hospital for Women and Children Da Nang Vietnam; ^6^ Department of Pediatrics, School of Medicine and Pharmacy The University of Da Nang Da Nang Da Nang Vietnam; ^7^ Alive & Thrive, Global Nutrition, FHI 360 Washington, DC USA

**Keywords:** breastfeeding, breastmilk expression, donor human milk, human milk bank, human milk donation, perceptions, Vietnam

## Abstract

Nutrition in early life plays a key role in shaping an infant's future health. There is limited understanding of the perspectives of Vietnamese mothers with children under 24 months of age regarding breastmilk expression, donation and use. In this cross‐sectional study, an online survey was administered through two parenting social media communities to assess opinions on breastmilk expression, breastmilk donation including contributions from bereaved mothers and the use of donor human milk. A 4‐point Likert scale was used to evaluate respondents' opinions, and demographic and breastfeeding information was collected. Among 375 respondents, almost 30% had received breastmilk from another woman, either through direct breastfeeding (14.7%), expressed breastmilk (12.5%) or from a human milk bank (2.7%). In this survey of 375 mothers, 84.0% indicated they would store excess breastmilk, while 75.7% and 69.6% would donate to a human milk bank or another mother, respectively. When faced with insufficient breastmilk, 88.5% of mothers would seek ways to increase supply, whereas 23.8% considered using commercial milk formula. Regarding milk expression among the 375 mothers, 78.4% preferred electric pumps, compared to 48.6% for manual pumps and 45.9% for hand expression. Additionally, 80.5% of the 375 mothers would suggest donating stored milk to bereaved peers and 85.6% would suggest mothers with mild COVID‐19 to continue breastfeeding with precautions. These findings indicate that this sample has positive views on breastfeeding, breastmilk donation and the use of donor human milk.

## Key messages

- There is limited understanding of the perspectives of potential human milk bank donors regarding breastmilk expression, donation and use in the context of LMICs such as Vietnam.
- Respondents, who were members of large, national, influential, evidence‐based parenting groups, held positive views on breastfeeding practices, breastmilk donation and the use of donor human milk.
- Future studies should explore the perspectives of fathers, close family members and health professionals.
- Using face‐to‐face interviews with a representative sample and conducting in‐depth interviews could be used to understand underlying reasons.

## Graphical abstract

In a Vietnamese online study, 375 mothers showed strong support for breastmilk donation and use, with many willing to donate excess milk and preferring electric pumps for expression. Most would increase their own supply before considering formula and advise breastfeeding with precautions during mild COVID‐19.

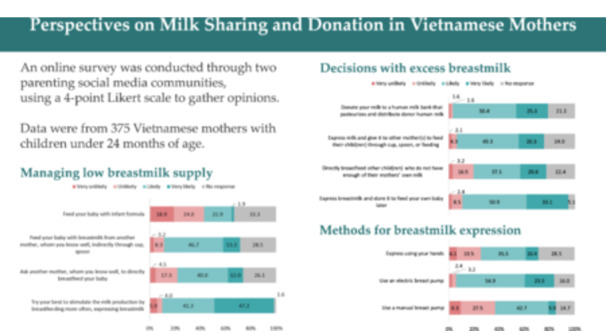

## 1 INTRODUCTION

Breastfeeding is the biological norm for feeding infants and is vital for the survival and development of infants and young children (Pérez‐Escamilla et al., 2023; WHO & UNICEF, 2020). Especially, the value of human milk for preterm and very low birthweight newborns (small vulnerable infants) is well established with strong evidence, and the World Health Organization (WHO) recommends donor human milk (DHM) from a human milk bank (HMB) as the best alternative for infants when a mother's own milk is not available (WHO Guidelines Review Committee, 2011; WHO, 2022; WHO & UNICEF, 2020). Since the first HMB opened in Austria over a century ago in 1909, there are now an estimated 756 HMBs operating in 66 countries (Haiden & Ziegler, 2016; Tableau, 2022; Tyebally Fang et al., 2021). The Global Alliance of Milk Banks and Associations (GAMBA) estimates that over 800,000 infants worldwide receive DHM annually (Shenker et al., 2021). However, globally, almost a million low‐birthweight infants without mothers' own milk do not have access to DHM (Shenker & Nangia, 2024).

Studies among mothers currently breastfeeding or those who have ever breastfed their children have shown that mothers are generally supportive of HMBs, although they may be more willing to donate breastmilk than to receive DHM (Ergin & Uzun, 2018; Iloh et al., 2018; Kimani‐Murage et al., 2019; Tu et al., 2022; Zhang et al., 2020). Factors such as age, religion, education, marital status and occupation have been associated with acceptance of HMBs (Ergin & Uzun, 2018; Iloh et al., 2018; Jackson & Obeng, 2022; Kimani‐Murage et al., 2019; Tu et al., 2022; Zhang et al., 2020). Women are more likely to donate if they are aware of HMBs, have heard about wet nursing, have positive attitude towards donating breastmilk or have had a child cared for on a neonatal unit (Gelano et al., 2018; Jackson & Obeng, 2022; Zhang et al., 2020).

Motivations for using milk from a peer included the health benefits and preference for human milk over commercial milk formula (CMF), especially when the children required breastmilk for optimal management or recovery from health problems (McCloskey & Karandikar, 2019) or as a temporary solution to the CMF shortage (Jackson & Obeng, 2022). The use of donor human milk provided relief, comfort and reduced mothers' self‐reported symptoms of postpartum depression and anxiety (McCloskey & Karandikar, 2019). However, they expressed concerns about safety and the accessibility to an HMB (Jackson & Obeng, 2022). Limited knowledge and misconceptions about milk banking may affect donation and use (Gelano et al., 2018; Ighogboja et al., 1995; Jahan et al., 2022; Magowan et al., 2020; Pal et al., 2019).

Two recent systematic reviews (Doshmangir et al., 2019; Gutierrez Dos Santos & Perrin, 2022) examining papers published before August 2020 explored the motivation of mothers to donate their breastmilk. They found that altruism and the desire to assist other babies were the primary social motivators for breastmilk donation, with a belief in the value of human milk and having an excess supply being key individual motivators. Support and encouragement from health professionals, other donor mothers and family members, along with knowledge of the donation process and its safety, facilitated donation. Reported barriers to donation included religious beliefs in certain cultures, a lack of knowledge about donation and time commitment. Additionally, education level was associated with the likelihood of donating milk to an HMB (Doshmangir et al., 2019; Gutierrez Dos Santos & Perrin, 2022). A recent paper in Vietnam revealed that mothers with higher education levels and from the community donated more breastmilk over an extended period than those with a lower education level and those who started donating breastmilk while in hospital (Tran, Nguyen, Nguyen, Barnett, et al., 2023).

Vietnam established its first HMB in 2017, and as of October 2023, it has five HMBs and three satellite HMBs in seven out of Vietnam's 63 provinces or cities. In 2021, the Vietnam Ministry of Health issued guidance and recommendations for HMBs, which hospitals can use to establish their own HMBs (Vietnam Ministry of Health, 2021). In Southeast Asia, the HMB network is expanding along with regional HMB guidelines through the Human Milk Banking Network in Southeast Asia (Human Milk Banking Network in Southeast Asia, 2021). To our knowledge, very little research has been undertaken on HMB in Southeast Asia in general and Vietnam in particular, where the HMB network is expanding. Several research questions arise during the expansion of the source of donors and recipients and the scaling‐up of the HMB network in Vietnam: What is the perception of using human milk from other women or from an HMB? What would women do if their own milk was insufficient or if they had surplus breastmilk? What are the common methods for expressing breastmilk? To contribute to answering these questions, we undertook an online survey which aimed to explore the perspectives on breastmilk expression, donation and use of own and donor milk with mothers with children aged 0–23 months, who were members of two social media communities focussed on parenting in Vietnam. Studying the perspectives of motivated members in influential, evidence‐based parenting groups will aid in understanding their potential as breastfeeding supporters, human milk bank donors and users. The insights gained can inform policymakers, managers and health staff who are promoting breastfeeding and scaling‐up sustainable HMBs.

## 2 SUBJECTS AND METHODS

### 2.1 Study design

This observational, cross‐sectional study employed a self‐administered online survey with mothers of children aged 0–23 months.

### 2.2 Setting

Vietnam is a lower‐middle‐income country, with a population size of more than 97.3 million and 37% of the population living in urban areas. Of the 1,567,000 infants born annually, 94% are born in a health facility and 8% are born with a low birthweight (UNICEF, 2021). The neonatal mortality rate of 10 per 1000 live births accounts for two‐thirds of infant mortality (16 per 1000 live births) and half of under‐5 mortality (20 per 1000 live births) (UNICEF, 2021). Internet and mobile use are common among young women with wide social media penetration (Kemp, 2023).

### 2.3 Participants, sample size and sampling

The survey intended for Vietnamese mothers aged 18 years or older who resided in Vietnam and had a child between 0 and 23 months of age. The focus on Vietnamese mothers aimed to create a more homogenous sample and facilitate the survey's implementation in the Vietnamese language. Additionally, certain questions were tailored to the mothers' experiences in Vietnam, making respondents living overseas less suitable for data collection. Limiting the child's age range to 0–23 months was done to minimize recall bias concerning breastfeeding practices.

The minimum sample size was 369, corresponding to a single‐group sample at a confidence level of 95%, a margin of error of 5% (Lwanga & Lemeshow, 1991) and anticipated population prevalence of ≤40% or ≥60% that were based on the values of most key breastfeeding behaviours and their behavioural determinants from previous studies in Vietnam (Doan et al., 2023; P. H. Nguyen et al., 2013; T. T. Nguyen et al., 2021). The participants were recruited through promoting the public link to the online survey to potential respondents through agreements with two parenting groups in Vietnam. With this sample size and sampling procedure, we did not attempt to generate representative findings for Vietnam or within the two parenting groups.

### 2.4 Variables and questionnaire development

For data collection, we employed an online structured questionnaire (Supplemental Material 1), which was based on Health Belief Model (HBM), Theory of Planned Behavior (TPB) and breastfeeding self‐efficacy (BSE) framework (Dennis & Faux, 1999; Lau et al., 2018; Salazar, 1991).

The cover page of this questionnaire provided participants with essential information, including the requirement that participants be at least 18 years of age, the purpose of the questionnaire, how the collected information would be utilized, an estimate of the time needed to complete the survey, the voluntary nature of participation and a statement assuring participants that identifiable information would not be collected. We also included a question to exclude women without a child under 24 months old, which also served to classify participants as mothers of infants aged 0–5 months, 6–11 months or 12–23 months. Survey questions specifically referred to the mother's youngest child.

The second page of the questionnaire consisted of seven components. The first five components assessed mothers' perceptions and views regarding (1) infant feeding decisions when faced with insufficient own breastmilk, (2) mothers' decisions with excess breastmilk, (3) likely methods to express breastmilk, (4) opinion for using breastmilk from bereaved mother and (5) opinion for using breastmilk of a mother who was infected with COVID‐19. These questions had responses gathered on a 4‐point Likert scale ranging from ‘very unlikely’ to ‘unlikely,’ ‘likely’ and ‘very likely’ to ensure user‐friendliness and compatibility on personal computers and mobile platforms. The questionnaire also collected information on (6) the mother's experience related to childbirth of the youngest child, including birth mode, birth weight, gestational age at birth, province of birth and the timing of breastfeeding initiation, as well as (7) background information such as age group, ethnicity and education level. The survey listed six specific provinces with an HMB or its services and offered the ‘other’ category which included the remaining 58 provinces. All participants received the same set of questions, which were organized in the same order (Supplemental Material 1).

We developed the initial English questionnaire, incorporating feedback from a panel of international and domestic experts. The enhanced English version was subsequently translated into Vietnamese to gather input from healthcare professionals and mothers in Vietnam. This feedback played a crucial role in refining the questionnaire. To facilitate data collection, we created online surveys using Microsoft Forms and securely hosted on an author's SharePoint platform, accommodating both English and Vietnamese questionnaires. These two online questionnaires underwent further testing by select mothers and experts for clarity, flow and ease to use. Then, the research team subsequently finalized the questionnaires, reset the count to zero and created a public link to the Vietnamese questionnaire.

### 2.5 Data collection

We contracted two parenting groups in Vietnam Betibuti (https://www.betibuti.com) and Toan Cau (https://vietsachhay.vn) to promote the public link to the survey to their members and to obtain 100‐200 completed questionnaires for each parenting group. In recognition of their services, we compensated each media group with a payment of $250. Betibuti is a large, evidence‐based parenting group with a closed national Fanpage boasting nearly 300,000 members, along with subgroups in almost all 64 provinces of Vietnam. Toan Cau has about 10,000 members. The members of these parenting groups include pregnant women and mothers of infants and young children, as well as influencers, people who join to support and those who have previously received support and now remain to support others.

These social media groups shared the survey link with their members and encouraged them to participate. Betibuti promoted the survey from 25 May to 6 June 2022, while Toan Cau promoted it from 1 to 18 July 022, spanning a total of 4 weeks. This link was for the cover page of the survey. Respondents who indicated that they did not have a child under 24 months old on the cover screening page were directed to the end of the survey where they were thanked and excluded. Participants who confirmed having a child under 24 months old and consented to participate were brought to the second page containing the main questions. Before clicking ‘submit’, the respondents were able to review and change their answers. After clicking ‘submit’, they received a thank you note, and their responses were stored in a password‐protected OneDrive. We did not perform completeness check given the respondents could skip any questions. In this online survey, we did not collect information on unique site visitors or unique visitors to the first page; we did not use cookies or IP checks.

### 2.6 Data management and analysis

Data were exported from Microsoft Form to an Excel file for further visualization and analysis. We employed the power pivot function within Microsoft Excel to summarize data and create relevant figures, with data presented in the tables below as rounded numbers (%). The descriptive statistics were from the sample of 375 mothers, with an option of no response in any statistics. We excluded participants who completed the survey before 150 s. The 150‐s threshold was based on the need to read approximately 550 words in the questionnaire (from the start of the main questions to the end) at an average adult reading speed of 220 words per minute (Brysbaert, 2019). The characteristics of the mothers whose records were excluded are similar to those retained for data analysis (Supplemental Material 2).

### 2.7 Ethical consideration

The study was conducted in accordance with the guidelines of the Declaration of Helsinki. It underwent review by the Institutional Review Board at FHI 360 (IRBNet ID 1898763‐1) and was classified as nonresearch in April 2022. A concise informed consent statement was presented on the screening page of the online assessment. Continuing with the survey was taken as indicating consent. There were no incentives offered (e.g., monetary, prizes or nonmonetary incentives such as an offer to provide the survey results). Collected data were securely stored on organization's SharePoint platform and only authorized to access by two authors.

## 3 RESULTS

### 3.1 Sample characteristics

In our study, of the 813 participants who completed the first page, 679 (83.5%) were women who had a child less than 24 months old and completed the survey. Of the 679 participants, we excluded 304 (44.8%) records where respondents completed the survey in questionably short time (<150 s). Information from 375 respondents was included in the analysis, with an average survey completion time of 6 min. Most respondents were between 25 and 34 years old, of Kinh ethnicity, and held a bachelor's degree or higher as their highest level of education (Table 1).

**Table 1:** Characteristics of mothers in the online survey.

Characteristics	% (*n* = 375)
Age (years)	
20–24	6.1
25–29	31.7
30–34	41.3
35–40	19.7
>40	1.1
Ethnicity	
Kinh	95.2
Other	4.5
No response	0.3
Highest level of education	
Less than secondary school (<12 years)	3.4
Secondary school (12 years)	8.0
Diploma from college (2–3 years after secondary school)	10.4
Bachelors from university (4–5 years after secondary school)	64.8
Masters or higher	11.7
No response	1.6

Table 2 presents the data on mothers' birth experiences and breastfeeding practices with their youngest child. Almost half (49.0%) of the 375 respondents gave birth in provinces with an HMB or its services: Ha Noi accounted for 27.7%, followed by Ho Chi Minh City at 14.4%, Da Nang at 3.2%, Can Tho at 1.9%, Quang Ninh at 1.3% and Quang Nam at 0.5%. Approximately, 47.5% of respondents gave birth in other provinces which did not have an HMB. The survey included 375 mothers of infants aged 0‐5 months (32%), 6‐11 months (30.4%) and 12–23 months (37.6%). Almost half (44%) of the 375 mothers gave cesarean births. Most of these respondents reported that their children had a birthweight between 2500 g and 3990 kg (90.1%) and were born at 37 weeks or later (92.3%).

**Table 2:** Birthplace, childbirth experience, child characteristics and breastfeeding practices.

Characteristics	% (*n* = 375)
Provinces/municipalities of childbirth	
Those with an HMB or its service	
Can Tho	1.9
Da Nang	3.2
Ha Noi	27.7
Ho Chi Minh City	14.4
Quang Nam	0.5
Quang Ninh	1.3
Other	47.5
No response	3.5
Child age	
<6 months	32.0
6–11 months	30.4
12–<24 months	37.6
Birth mode	
Cesarean birth	44.0
Vaginal birth	54.9
No response	1.1
Birthweight	
Less than 2500 g	5.1
Between 2500 to 3990 g	90.1
More than or equal to 4000 g	4.0
No response	0.8
Termed birth (at 37 weeks or more)	
Yes	92.3
No	6.1
No response	1.6
Breastfeeding initiation from time of birth	
Within the first hour (<60 min)	50.4
1 h or more (≥60 min)	45.3
No response	4.3
Fed breastmilk from another woman	
No	70.4
Yes, directly breastfed by another woman	14.7
Yes, fed expressed breastmilk from another woman	12.5
Yes, from a human milk bank	2.7
Breastfed on the previous day	
Yes	90.7
No	7.5
No response	1.9

Around half of the 375 mothers stated that their child breastfed within the first hour of birth (50.4%) and over 90% reported that their child had breastfed the day before completing the survey. Also, there was almost 30% of babies who had received breastmilk from another woman: 14.7% reported direct breastfeeding by another woman, 12.5% fed expressed breastmilk from another woman and 2.7% used breastmilk from an HMB (Table 2).

### 3.2 Infant feeding decisions when faced with insufficient breastmilk supply

**Figure 1:**
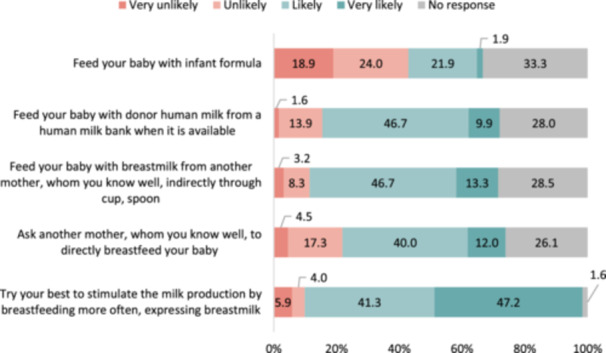
Infant feeding decisions when faced with insufficient own breastmilk (*n* = 375). *Question 2*. If you had a 15‐day‐old newborn and thought that you did not have sufficient milk, how likely would you be to….

Figure 1 depicts mothers' likelihood of selecting various infant feeding methods if they experienced an insufficient breastmilk supply for their 15‐day‐old newborn. For most (88.5%) of the 375 mothers, it was very likely or likely that they would opt for a solution to increase their breastmilk supply if they perceived an insufficient milk supply. In this sample, the use of breastmilk from another mother was marked as a likely or very likely choice: 56.6% of respondents favoured using donor milk from an HMB, 60.0% preferred using expressed milk from a mother known to them and 52% were in favour of asking a mother known to them to directly breastfeed their baby. The use of breastmilk from another mother or from an HMB was considered acceptable. However, 23.8% of the 375 mothers who responded to this question found feeding CMF to be a very likely or likely choice.

### 3.3 Mothers' decisions with excess breastmilk

**Figure 2:**
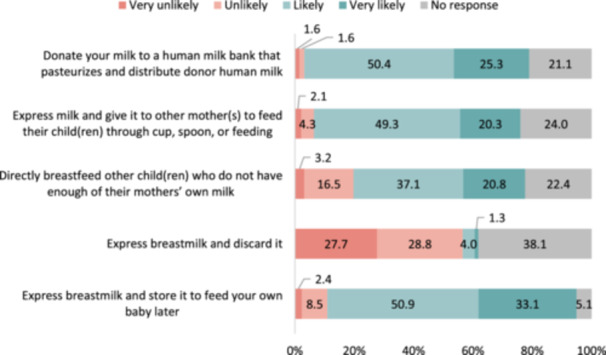
Mothers' decisions with excess breastmilk (*n* = 375). *Question 3*. If you have surplus/extra breastmilk, how likely would you….

Figure 2 focusses on mothers' decisions regarding surplus breastmilk, including their likelihood to donate, discard, indirectly or directly breastfeed and store. The majority of the 375 mothers indicated that they were likely or very likely to express and store their breastmilk for their own baby at a later time (84.0%). Additionally, a significant portion reported that they would be likely to donate their breastmilk to an HMB (75.7%) or provide it to another mother for her child (69.6%). In this sample of 375 mothers, the likelihood of directly breastfeeding other children was somewhat lower, with 20.8% responding as very likely and 37.1% as likely; and relatively few mothers indicated they were likely or very likely to discard their surplus breastmilk (5.3%).

### 3.4 Likely method for breastmilk expression

**Figure 3:**
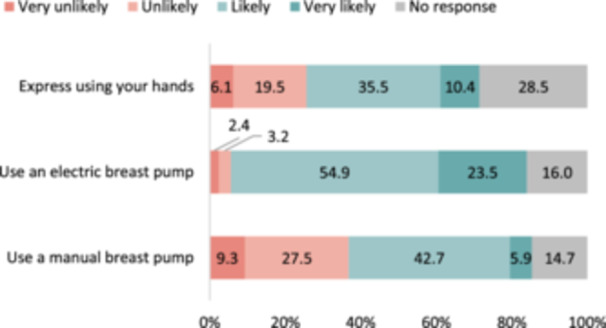
Likely methods to express breastmilk (*n* = 375). *Question 4*. To express milk for your child or other children, how likely would you….

Figure 3 presents mothers' likelihood to express breastmilk using different methods, specifically by hand, with an electric breast pump and with a manual breast pump. For the 375 mothers, the most common method, chosen as ‘very likely’ or ‘likely,’ was using an electric breast pump, selected by 78.4% of the respondents, which was followed by using a manual breast pump (48.6%) and hand expression (45.9%).

### 3.5 Breastfeeding mothers and bereaved mothers

**Figure 4:**
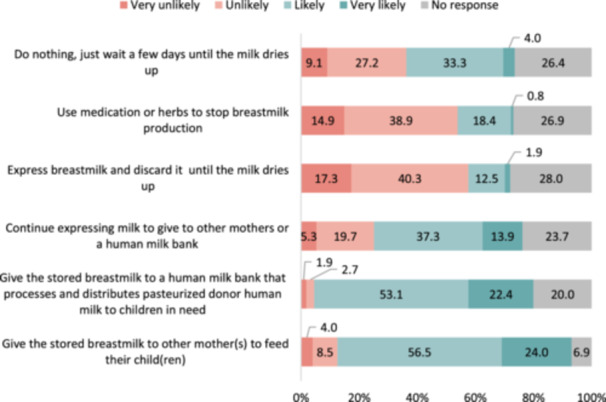
Opinion for using breastmilk from bereaved mother (*n* = 375). *Question 5*. For a mother whose child passed away, how likely would you suggest her to….

Figure 4 depicts mothers' likelihood to suggest specific infant feeding practices to a bereaved mother. In the sample of 375 mothers, a significant number indicated that they were ‘very likely’ or ‘likely’ to suggest giving their stored breastmilk to other mothers (80.5%) and to an HMB (75.5%). Additionally, a substantial proportion (51.2%) would suggest continuing expressing breastmilk for other babies and for donation to an HMB. Regarding methods to stop breastmilk production among the 375 mothers, respondents were inclined to suggest doing nothing and waiting for the breastmilk to naturally dry up (37.3%), using medication or herbs to stop breastmilk production (19.2%) and expressing and discarding breastmilk until it dries up (14.4%).

### 3.6 Mothers' opinions on the breastmilk of mothers with COVID‐19

**Figure 5:**
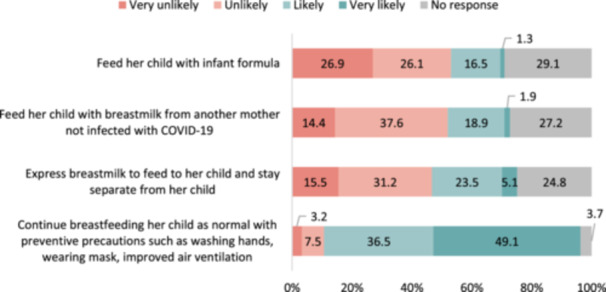
Opinion for using breastmilk of a mother who was infected with COVID‐19 (*n* = 375). *Question 6*. If a mother of a four‐month‐old baby is infected with COVID‐19 with mild symptoms, how likely would you suggest that she….

Figure 5 illustrates respondents' likelihood to suggest certain infant feeding practices to a mother of a 4‐month‐old baby experiencing mild COVID‐19 symptoms. In the sample of 375 mothers, when it came to the likelihood of suggesting that the mother with COVID‐19 should continue to breastfeed her child as normal while taking preventive precautions, a combined total of 85.6% of mothers indicated they were likely (36.5%) or very likely (49.1%). In contrast, about half of the 375 mothers were unlikely or very unlikely to suggest feeding their child with breastmilk from a mother without COVID‐19 (53%) or expressing breastmilk to feed to their child while staying separate from the child (46.7%).

## 4 DISCUSSION

This online, self‐administered exploratory study aimed to investigate the perspectives of mothers with a child aged 0–23 months regarding breastmilk expression, donation and usage among mothers in two parenting social media communities in Vietnam. The study revealed that mothers were inclined to breastfeed their own children, stimulate breastmilk production when they perceived a shortage of breastmilk, use breast pumps for breastmilk expression and were open to both donating and using DHM.

### 4.1 Women breastfeeding their own children

Approximately, half of the 375 surveyed mothers reported that their child had breastfed within the first hour of birth (50.4%), a rate significantly higher than the national estimate of 23.5% (Vietnam General Statistics Office & UNICEF, 2021) and in three provinces, where the rate was 39.7% (Nguyen et al., 2022). In this sample, a striking 90% of mothers with children under 24 months breastfed their children on the previous day, surpassing the rates observed in previous studies. While a direct comparison was challenging, it is evident that the prevalence in this sample exceeded the national estimate. According to the national estimate, continued breastfeeding at 12–15 months was 66.5%, and at 20–23 months, it was 23.2% (Vietnam General Statistics Office & UNICEF, 2021). This high level of breastfeeding suggests that the research attracted women who were more committed to breastfeeding than average and more likely to be potential human milk bank donors. In this study, we found that women, assuming they have surplus breastmilk, are likely to express and store it for future use. This is common when mothers need to return to work or anticipate having less breastmilk available later.

Systematic reviews have shown that perceived breastmilk insufficiency is common and is negatively associated with exclusive and continued breastfeeding (Huang et al., 2022). However, our findings revealed that women would opt for a solution that continued breastfeeding or to use DHM from an HMB if available, rather than using CMF if they had insufficient breastmilk supply. This finding is encouraging, although it appears to be higher than the population estimate. For instance, a prior study in Vietnam (*n* = 726) showed a significant number of women bringing CMF to maternity facilities they gave birth (44.9%), purchasing CMF (35.4%) within or near the facility or both bringing and purchasing (7.6%) CMF (Nguyen et al., 2021). This suggests the prevailing norm of perceived breastmilk insufficiency for newborns in their first few days of life and that CMF is a solution. Social norms, self‐efficacy and the practice of early and exclusive breastfeeding were negatively associated with perceived breastmilk insufficiency and related breastfeeding practices (Huang et al., 2022; Nguyen et al., 2016). Previous studies have demonstrated that participation in social media can impact breastfeeding practices and norms (Morse & Brown, 2022; Orchard & Nicholls, 2022). Being members of parenting support groups, such as Betibuti, the participants in our study may have more positive attitudes towards breastfeeding as well as higher self‐efficacy and peer support and thus a higher prevalence of using recommended breastfeeding practices and of continuing breastfeeding for a longer time (Baxter & Lawton, 2022).

### 4.2 Women and breastmilk sharing

The use of breastmilk from other mothers has a long history and remains relevant today (Stevens et al., 2009). WHO also recommends using breastmilk from other women if a mother's own milk is unavailable (WHO, & UNICEF, 2003). In this sample of 375 mothers, we found that almost 30% of the women had fed their child breastmilk from another woman: 14.7% reported direct breastfeeding by another woman, 12.5% fed expressed breastmilk from another woman and 2.7% used milk from an HMB. A previous descriptive, cross‐sectional study with 726 mothers of infants in Vietnam showed that only 4.4% of children used breastmilk from other women in the first 3 days of life (Nguyen et al., 2022). We were unable to find published data on the national prevalence of wet nursing or informal breastmilk sharing. However, some studies suggest that informal breastmilk sharing is increasingly popular in other countries (Ergin & Uzun, 2018; Konukbay et al., 2023; Kuznetsova et al., 2020; Marinelli, 2020; McCloskey & Karandikar, 2019; Obeng et al., 2022). An online study in the US showed that 29.1% of mothers with low milk supply reported using breastmilk from another mother for their infants, and this usage helped increase the continued breastfeeding rate at 6 months (Cassar‐Uhl & Liberatos, 2018). Being members of a closed support group can foster trust among members (e.g., those with similar characteristics and norms, who know one another) and can reduce the fear of milk‐sharing stigma and health risks, thereby encouraging more informal milk‐sharing within the group (Gribble, 2018; Jackson & Obeng, 2022; Schafer et al., 2018).

The women in this study were commonly willing to share their breastmilk with other children in need or donate it to an HMB if available. This included stored breastmilk, especially when their own children no longer require it (Tran, Nguyen, Nguyen, Barnett, et al., 2023). Although we did not collect views about potential risks relating informal breastmilk sharing, mothers in previous studies in other countries indicated concerns that this practice can carry potential risks of infectious diseases or contamination due to substances ingested by the donor mother, suboptimal storage conditions or the introduction of other liquids (Kuznetsova et al., 2020; Lubbe et al., 2019). Therefore, it is essential to carefully evaluate the benefits and risks of breastmilk sharing and make informed decisions that prioritize the health and well‐being of the baby (Marinelli, 2020). There are also concerns related to the bonding of the children with the donors; the child might inherit characteristics from the donors (e.g., temperament, intelligence, language accent) or establish kinship relationships with the donors (Gribble, 2018; Khalil et al., 2016; Lubbe et al., 2019). Securing DHM and the fear of running out of it were reported as stressors among mothers who received DHM (McCloskey & Karandikar, 2019). A qualitative documentary analysis, examining 111 media articles mostly from the United States, United Kingdom and Australia, reveals concerns related to the lack of regulation, potential commercialization, contamination of DHM and potential poor lifestyle factors (e.g., alcohol, smoking, drugs, personal hygiene) of the donors (Dowling & Grant, 2021). Some articles also highlighted that milk sharing was often portrayed as dirty, sexualized or risky (Dowling & Grant, 2021). Yet, women who seek milk from other mothers seek donor milk because they want sometime better than CMF feeding and undertake risk mitigation strategies (Gribble, 2014a, 2014b).

Despite the expansion of HMB networks, numerous unknown factors persist. Not all those who are willing to share milk directly with another mother will be willing or able to donate to an HMB, if there is no local milk bank or if they do not meet the criteria for donors or for other reasons (Gribble, 2013; Shenker et al., 2024). Globally, the majority of HMBs lack routine national regulation and global guidelines (Tyebally Fang et al., 2021). This lack of regulation can result in potential consequences, including the overuse of DHM instead of investing in supporting mothers in establishing and maintaining their breastmilk and directly breastfeed (Shenker & Nangia, 2024; Tran et al., 2021). It may also put donor mothers at risk of exploitation and lead to the commercialization of DHM (Shenker & Nangia, 2024; Tyebally Fang et al., 2021). Gathering perceptions of the risks among the parents and those who influence them in this setting would be an important study for the expansion of HMB.

### 4.3 Women's views on bereaved women donating breastmilk and in assisting those with COVID‐19 to continue breastfeeding their children

In this study, for bereaved mothers, the primary advice was to donate stored breastmilk to an HMB or to other mothers in need. Some women were likely to suggest to a bereaved mother that she continue expressing breastmilk for donation to an HMB or to other mothers. A recent study found that few bereaved women become breastmilk donors at an HMB in Vietnam (Tran, Nguyen, Nguyen, Mathisen, et al., 2023). The main reason is that HMBs typically have an adequate supply of DHM from other women to meet the needs of newborns in their hospitals and even in neighboring provinces. As a result, they do not typically request breastmilk from bereaved mothers. Additionally, there are concerns about the potential psychological harm to the donating women, such as triggering the pain of their loss (Tran, Nguyen, Nguyen, Mathisen, et al., 2023). In cases of bereavement, the mothers were more likely to take an active role in approaching HMBs to donate breastmilk rather than health workers suggesting donation (Tran, Nguyen, Nguyen, Mathisen, et al., 2023). A qualitative study of seven bereaved women in Northern Ireland found three themes related to bereavement and breastmilk donation: (1) fulfilling the mother role; (2) the power of being able to ‘do’ and (3) making good from the bad (Ward et al., 2023). However, four out of the seven women ceased donating after their children's deaths (Ward et al., 2023).

If a mother with COVID‐19 had mild symptoms, the main suggestion was to continue breastfeeding as usual while taking preventive precautions. This finding aligns with WHO and national guidance for women with COVID‐19, emphasizing the importance of adhering to safety precautions if they wish to breastfeed (Gribble et al., 2022). During the survey planning period, Vietnam had a high prevalence of existing and new COVID‐19 cases, low vaccine coverage, common concerns about transmission and a lack of available global and national guidelines (Our World Data, 2023; Wesolowska et al., 2022). However, by the time of data collection, COVID‐19 was under control with few new cases, mostly mild symptoms and almost no deaths due to COVID‐19 (Our World Data, 2023). Also, guidelines from WHO and Vietnam were available, which supported breastfeeding during COVID‐19 (Gribble et al., 2022). Heightened awareness of the guidance may have contributed to supportive responses to this survey question.

### 4.4 Women's use of electric breast pumps

We found that the most common methods of breastmilk expression were electric breast pumps, followed by manual breast pumps and hand expression. A Cochrane Library review (Becker et al., 2016) demonstrated that there were no clinically significant differences between these methods in terms of milk contamination, maternal breast or nipple pain or damage, breastmilk volume or the rate of increasing volume after using any expression method. While there were some slight variations in nutrient quality, the energy content remained similar (Becker et al., 2016). Hand expression of milk is a minimal cost method available worldwide, particularly in situations where electricity may be lost and sanitation poor such as in emergencies (Becker, 2021; IFE Core Group, 2017). However, there is a transformation in breastfeeding practices with the use of electric breast pumps, which has potential harms for both mothers and infants (Rasmussen & Geraghty, 2011). In addition, there are environmental impacts of using breast pumps associated with manufacturing and disposal, energy consumption for its use as well as for cleaning and sterilization of the pumps, bottles and teats. Milk storage and use often requires plastic bags or containers, which adds to plastic use and waste (Becker, 2021; Meier et al., 2016; Rasmussen & Geraghty, 2011).

The breast pump market in Vietnam is large and rapidly expanding. According to import data obtained as of August 5, 2023, from 70 countries, Vietnam ranks as the world's third‐largest importer of breast pumps, trailing only India and the United States (Volza Grow Global, 2023). A variety of breast pumps are available in Vietnam, with prices of high‐end models ranging from 270 to 420 USD (Vo, 2021). Limited time and skill of health workers to assist mothers learning how to hand express, combined with extensive marketing of high‐cost electric pumps, may contribute to the preference for electric pumps, which may not always align with the best interests of mothers and families (Becker et al., 2016). Indeed, a previous study in Vietnam revealed that due to heavy workloads, healthcare workers were unable to allocate sufficient time for breastfeeding counselling and support (Nguyen et al., 2021). Meanwhile, industry representatives were present in hospitals, actively promoting their products, which could potentially influence women to choose their products (Nguyen et al., 2021). Women should be made aware of methods of expressing breastmilk, their costs, risks, benefits and environmental impacts to make informed decisions and receive guidance free of commercial interest. The mothers should be well informed that direct breastfeeding should be prioritized, and expressed milk is a feasible way to provide human milk when direct nursing is not possible due to temporary or ongoing separation of the mother and child, extremely premature birth or illness (Becker, 2021; Meier et al., 2016; Rasmussen & Geraghty, 2011).

### 4.5 The implication of positive deviance

We believe that studying the perspectives of highly motivated and influential mothers, often referred to as “positive deviance,” is a strength of this study. Positive deviance, an approach that identifies and learns from individuals or groups who achieve better outcomes than their peers despite similar challenges, has significant implications for breastfeeding practices (Baxter & Lawton, 2022). In this study sample, the respondent mothers were more likely to breastfeed their own children over a longer period than the population average and to be willing to support other women including milk sharing. Examining positive deviance, an approach that identifies and learns from individuals or groups who achieve better outcomes than their peers despite similar challenges, has significant implications for developing a supportive environment that fostering social and behavioural change towards improving breastfeeding practices (Baxter & Lawton, 2022).

### 4.6 Limitations

Our study has some limitations. First, our sample was recruited via two large parenting groups, and there may be a selection bias, with mothers more committed to breastfeeding being more likely to take part in this survey and to respond positively to breastfeeding. Due to budget constraints, we were unable to promote the survey to a larger and more diverse population or to recruit more participants randomly to generalize the findings. In addition, because our survey was only available online and within these two parenting groups, it might have attracted more respondents who have higher income and education levels, belong to the ethnic majority group and live in urban areas (Jackson & Obeng, 2022; UNICEF, 2021). The impact of accessing a mobile device or computer and the internet may be minimal since most Vietnamese adults, especially the younger generation, have access to the internet and smart devices and are members of social media networks (Kemp, 2023). Without attempting to generalize these descriptive findings even within the social media groups, we did not perform any statistical corrections, such as weighting of items or propensity score adjustments to account for the nonrepresentative sample.

Second, response rate could be an issue because respondents had the option to skip questions they did not want to answer or stop responding to the survey. Recognizing this potential limitation, we used a structured questionnaire and reduced the survey's length. We also used a Likert scale that respondents could easily understand and is similar to ratings on the internet and social media.

Third, in this study, we were not able to explore the reasons behind the data. It was not feasible for this online survey (e.g., time required from the participants) to include more in‐depth questions, including open‐ended questions. We did not track respondents, which meant we couldn't follow‐up with specific participants for more in‐depth responses. Face‐to‐face interviews were not planned because it was during the COVID‐19 pandemic, making in‐person meetings challenging. We did not gather information from other caregivers or health workers, who would influence the perception and decisions of the mothers. Though most of the research found explored the views of mothers, views of other key people are also influential in willingness to donate or to use DHM, such as grandmothers and fathers (Mondkar et al., 2018). The decisions to start and continuing to donate breastmilk are personal and affected by factors relating to the women, her family, community, the hospitals and health workers and media (Kaech et al., 2022; Zhang et al., 2020). Understanding the perspective of the mothers is important to maximize the use of breastmilk for their own children and donation of breastmilk to an HMB.

Future studies should explore the perspectives of fathers and close family members such as grandmothers who may influence breastmilk donation and usage. Additionally, gaining insights from health professionals who provide advice to mothers and families in situations involving donor human milk would be valuable. Conducting face‐to‐face interviews with a representative sample and conducting in‐depth interviews with key informants and caregivers could provide more in‐depth information. Future studies and guidelines will be needed to learn more about milk expression methods, wet nursing and milk sharing.

## 5 CONCLUSION

Our findings indicate that Vietnamese mothers of children aged 0–23 months in this sample of mothers in two popular social media communities on parenting in Vietnam support breastfeeding, breastmilk donation and the use of donor human milk. The findings from this study contribute to our understanding of certain cultural and societal factors that influence the acceptance and utilization of donor breastmilk among members of influential, evidence‐based parenting groups. These insights are valuable for comprehending the high potential of these mothers to donate and to use donor human milk, and this knowledge can be disseminated to other parenting groups and beyond. The identified knowledge can inform policy development and interventions relating to breastfeeding, DHM and HMB.

## AUTHOR CONTRIBUTIONS

*Conceptualization*: Tuan T. Nguyen, Ngoc L. Huynh, Genevieve Becker, Hoang T. Tran, Jennifer Cashin, Roger Mathisen. *Methodology*: Tuan T. Nguyen, Ngoc L. Huynh, Genevieve Becker, Jennifer Cashin. *Formal analysis*: Tuan T. Nguyen. *Validation*: Tuan T. Nguyen, Ngoc L. Huynh. *Investigation*: Tuan T. Nguyen. *Resources*: Roger Mathisen. *Data curation*: Tuan T. Nguyen. *Drafting the manuscript*: Tuan T. Nguyen, Ngoc L. Huynh, Genevieve Becker. *Review and editing*: Tuan T. Nguyen, Ngoc L. Huynh, Genevieve Becker, Hoang T. Tran, Jennifer Cashin, Roger Mathisen. *Visualization*: Tuan T. Nguyen. *Supervision*: Tuan T. Nguyen. *Project administration*: Tuan T. Nguyen. *Funding acquisition*: Roger Mathisen. All authors have read and agreed to the published version of the manuscript.

## CONFLICT OF INTEREST STATEMENT

The authors declare no conflict of interest.

## Supporting information

Supporting information.

## Data Availability

The data that support the findings of this study are openly available in Harvard Dataverse at https://doi.org/10.7910/DVN/XOJRRH.
