# Supplementary material for: Views on wet nursing and expressing breastmilk for sharing and human milk bank donation among mothers in two parenting social media communities in Vietnam
Source: Matern Child Nutr. 2024 Aug 15;21(1):e13694. doi: 10.1111/mcn.13694 (PMC11650063; doi:10.1111/mcn.13694)
Supplement: Supplementary file 1 — Supporting information. [file MCN-21-e13694-s001.pdf]

Supplemental Material 1

SM 1.1. Survey questionnaire in English

I. COVER PAGE:

Perspectives on breast milk of mothers of children under 24 months of age in Vietnam

You are invited to take part in an online poll that lasts about 5-10 minutes. This survey is for Vietnamese mothers aged 18 and older and gave birth within the last 24 months.

We would like to hear about your views on 1) expressing and donating breast milk and 2) using donor breast milk. The information will be used to inform activities to improve child feeding practices in Vietnam.

Your participation is voluntary and you may omit certain questions that you do not wish to answer. We will not collect any identifying information. If you have any questions, please contact us at PI_email_address@fhi360.org.

1. What is the age of your youngest child?

1. Less than 6 months (<180 days)
2. 6 months to less than 12 months (180-365 days)
3. 12 months to less than 24 months (366-730 days)
4. Does not have a child, or the child is above two years of age (Not eligible)-> Thank you page.

II. QUESTION PAGE:

Notes:

The questions 2-6 are on a scale from Very unlikely, Unlikely, Likely, to Very likely.

A human milk bank (HMB) is a service established to recruit breast milk donors, collect donated milk, and then pasteurize, screen, store, and distribute the milk to meet infants' specific needs for optimal health.

2. If you had a 15-day-old newborn and thought that you did not have sufficient milk, how likely would you ...

- Try your best to stimulate milk production by breastfeeding more often, expressing breastmilk, eating special foods
- Ask another mother, whom you know well, to directly breastfeed your baby
- Feed your baby with breastmilk from another mother, whom you know well, indirectly through a cup, spoon, or bottle
- Feed your baby with donor human milk from *a human milk bank* when it is available
- Feed your baby with infant formula

3. If you have surplus/extra breastmilk, how likely would you …

- Express breastmilk and store it to feed your own baby later
- Express breastmilk and discard it
- Directly breastfeed other child(ren) who do not have enough of their mothers' own milk
- Express milk and give it to other mother(s) to feed their child(ren) through cup, spoon, or feeding bottle
- Donate your milk to a human milk bank that pasteurizes and distributes donor human milk to children in need

4. To express milk for your child or other children, how likely would you …

- Use a manual breast pump
- Use an electric breast pump
- Express using your hands

5. For a mother whose child passed away, how likely would you suggest her to …

- Give the stored breastmilk to other mother(s) to feed their child(ren)
- Give the stored breastmilk to a human milk bank that processes and distributes pasteurized donor human milk to children in need
- Continue expressing milk to give to other mothers or a human milk bank
- Express breastmilk and discard it / throw it away until the milk dries up
- Use medication or herbs to stop breastmilk production
- Do nothing, just wait a few days until the milk dries up

6. If a mother of a four-month-old baby is infected with COVID-19 with mild symptoms, how likely would you suggest that she…

- Continue breastfeeding her child as normal with preventive precautions such as washing hands, wearing a mask, and improved air ventilation
- Express breastmilk to feed her child and stay separate from her child
- Feed her child with breastmilk from another mother not infected with COVID-19
- Feed her child with infant formula

*7. **Birth mode*** of your youngest child

- Cesarean birth
- Vaginal birth

8. What was your youngest child's birthweight?

- Less than 2.5 kg
- Between 2.5 to 3.99 kg
- More than or equal to 4.0 kg

9. Was your youngest child born term (at 37 weeks or longer)?

- Yes
- No

10. The province where you gave birth?

- Da Nang
- HCM City
- Ha Noi
- Quang Ninh
- Quang Nam
- Can Tho
- Other

11. How soon after birth did you put the youngest child to the breast to feed her/him for the first time?

- Within the first hour (< 60 min)
- One hour or more (≥ 60 min)
- I have never breastfed my child

12. Has your youngest child ever been fed breastmilk from another woman?

- No
- Yes, directly breastfed by another woman
- Yes, fed expressed breastmilk from another woman
- Yes, from a human milk bank

13. Did you breastfeed your youngest child yesterday?

- Yes
- No

Could you tell a little bit more about yourself? (One choice)

14. Your age:18-19; 20-24; 25-29; 30-34; 35-40, over 40

15. Your ethnicity: 1) Kinh; 2) Other

16. (if Other) Please type the name of your ethnicity below
(______________)

17. What is the highest level of education you have completed?

- Less than junior secondary school (≤ 9 years)
- Junior secondary school (10-11 years)
- Secondary school (12 years)
- Diploma
- Bachelors,
- Masters, or higher

III. THANK YOU

SM 1.2. Survey questionnaire in Vietnamese (local language)

I. TRANG BÌA:

Bạn được mời tham gia một cuộc thăm dò ý kiến trực tuyến kéo dài khoảng 5-10 phút. Cuộc thăm dò này dành cho các bà mẹ Việt Nam (từ 18 tuổi trở lên) đã sinh con trong vòng 2 năm qua (24 tháng).

Chúng tôi muốn tìm hiểu về quan điểm của bạn về 1) vắt và hiến tặng sữa mẹ và 2) việc sử dụng sữa mẹ của người hiến tặng. Thông tin sẽ được sử dụng để thông báo các hoạt động cải thiện thực hành cho ăn cho trẻ em ở Việt Nam.

Sự tham gia của bạn là tự nguyện và bạn có thể bỏ qua một số câu hỏi nhất định mà bạn không muốn trả lời. Chúng tôi sẽ không thu thập bất kỳ thông tin nhận dạng nào. Nếu bạn có thắc mắc, hãy liên hệ với chúng tôi theo email tnguyen@fhi360.org.

1. Con út của bạn bao nhiêu tuổi?

5. Dưới 6 tháng (<180 ngày)
6. 6 tháng đến dưới 12 tháng (180-365 ngày)
7. 12 tháng đến dưới 24 tháng (366-730 ngày)
8. Không có con, hoặc đứa trẻ trên hai tuổi (Không đúng đối tượng) -> Xin trân thành cảm ơn.

II. TRANG CÂU HỎI:

Ghi chú:

Các câu hỏi 2-6 nằm trên thang điểm từ 1-Hoàn toàn không đồng ý, 2-Không đồng ý, 3-Đồng ý, 4) Hoàn toàn đồng ý.

Ngân hàng sữa mẹ (HMB) là một dịch vụ được thành lập để vận động bà mẹ hiến tặng sữa, thu thập sữa hiến tặng, sau đó thanh trùng, sàng lọc, lưu trữ và phân phối sữa để đáp ứng nhu cầu cụ thể của trẻ sơ sinh về sức khỏe tối ưu.

2. Nếu con bạn 15 ngày tuổi và bạn nghĩ rằng mình không có đủ sữa, bạn sẽ ...

- Cố gắng hết sức để kích thích sản xuất sữa bằng cách cho con bú thường xuyên hơn, vắt sữa, ăn các loại thực phẩm đặc biệt
- Cho con bú trực trực tiếp từ một người mẹ khác, người mà bạn biết rõ
- Xin sữa từ một người mẹ mà bạn biết rõ và cho uống bằng cốc, thìa hoặc bình sữa
- Cho bé uống sữa hiến tặng từ ngân hàng sữa mẹ (nếu có)
- Cho bé uống sữa công thức

3. Nếu bạn có dư thừa sữa mẹ, bạn sẽ ...

- Vắt sữa và lưu trữ nó để nuôi con trong thời gian tới
- Vắt bỏ sữa
- Cho những đứa trẻ khác (mà mẹ chúng không đủ sữa) bú trực tiếp
- Vắt sữa và đưa cho người mẹ khác để cho con ăn qua cốc, thìa hoặc bình
- Tặng sữa của bạn cho một ngân hàng sữa mẹ

4. Để vắt sữa cho con bạn hoặc những đứa trẻ khác, bạn sẽ ...

- Dùng máy vắt sữa bằng tay
- Dùng máy vắt sữa chạy điện hay pin
- Dùng tay

5. Một người mẹ có con qua đời, bạn sẽ khuyên cô ấy ...

- Tặng sữa mẹ đã vắt và đang được trữ đông cho những đứa trẻ khác
- Tặng sữa mẹ đã vắt và đang được trữ đông cho ngân hàng sữa mẹ
- Tiếp tục vắt sữa để tặng cho các bà mẹ khác hoặc ngân hàng sữa mẹ
- Vắt bỏ sữa mẹ cho đến khi ngừng tiết sữa
- Sử dụng thuốc hoặc thảo mộc để ngừng tiết sữa
- Không làm gì cả, chỉ cần đợi vài ngày cho đến khi ngưng tiết sữa

6. Nếu mẹ của một em bé bốn tháng tuổi bị nhiễm COVID-19 với các triệu chứng nhẹ, bạn sẽ khuyên cô ấy ...

- Tiếp tục cho con bú như bình thường với các biện pháp phòng ngừa lây nhiễm như rửa tay, đeo khẩu trang, cải thiện thông gió
- Vắt sữa mẹ để con ăn và cách ly con
- Cho con ăn sữa mẹ từ một người mẹ khác không bị nhiễm COVID-19
- Cho trẻ ăn sữa công thức

7. Bạn sinh cháu út theo cách nào?

- Sinh mổ
- Sinh thường

8. Lúc sinh, cháu út của bạn nặng bao nhiêu cân?

- Dưới 2,5 kg
- Từ 2,5 đến dưới 4,0 kg
- Nhiều hơn hoặc bằng 4,0 kg

9. Bạn sinh cháu út có đủ tháng không (ở tuần thứ 37 hoặc nhiều hơn)?

-Có

-Không

10. Bạn sinh con ở tỉnh nào?

- Đà Nẵng
- TP Hồ Chí Minh
- Hà Nội
- Quảng Ninh
- Quảng Nam
- Cần Thơ
- Khác

11. Sau khi sinh bao lâu bạn bắt đầu cho cháu bú lần đầu tiên?

- Trong vòng một giờ đầu tiên (< 60 phút)
- Một giờ trở lên (≥ 60 phút)
- Tôi chưa bao giờ cho con bú sữa mẹ

12. Con út của bạn đã bao giờ được cho ăn sữa mẹ từ một người mẹ khác chưa?

- Chưa
- Có, bú trực tiếp một người phụ nữ khác
- Có, cho ăn sữa mẹ từ một người phụ nữ khác vắt cho
- Có, cho ăn sữa từ một ngân hàng sữa mẹ

13. Ngày hôm qua con út của bạn có bú sữa mẹ không?

- Có
- Không (kể cả trường hợp chưa bao giờ được bú mẹ)

14. Độ tuổi của bạn:18-19; 20-24; 25-29; 30-34; 35-40, trên 40

15. Dân tộc của bạn: 1) Kinh; 2) Khác

16. (nếu khác) Vui lòng nhập tên dân tộc của bạn dưới đây
(______________)

17. Trình độ học vấn cao nhất mà bạn đã hoàn thành là gì?

- Ít hơn trung học cơ sở (≤ 9 năm)
- Trung học cơ sở (10-11 năm)
- Trung học cơ sở (12 năm)
- Trung, sơ cấp
- Đại học
- Sau đại học

III. CẢM ƠN BẠN ĐÃ THAM GIA TRẢ LỜI CÂU HỎI

Supplemental Material 2.

Sensitivity analysis: Comparing characteristics of mothers responded within 2.5 minutes (excluded) and longer (included)

	Excluded<br>%<br>(n = 304)	Included<br>%<br>(n = 375)	Total<br>%<br>(n = 679)
Age (y)	304	375	
18-19	1.3	0.0	0.6
20-24	2.3	6.1	4.4
25-29	17.1	31.7	25.2
30-34	23.0	41.3	33.1
35-40	43.8	19.7	30.5
> 40	11.8	1.1	5.9
No response	0.7	0.0	0.3
Ethnicity			
Kinh	98.4	95.2	52.6
Other	1.0	4.5	2.9
No response	0.7	0.3	0.4
Highest level of education			
Less than junior secondary school (≤ 9 years)	1.6	1.3	1.5
Junior secondary school (10-11 years)	2.0	2.1	2.1
Secondary school (12 years)	5.9	8.0	7.1
Diploma from college (2-3 years after secondary school)	9.9	10.4	10.2
Bachelors from university (4-5 years after secondary school)	73.4	64.8	68.6
Masters or higher	5.9	11.7	9.1
No response	1.3	1.6	1.5
